# Cultural topography of publicness: Assessment of the publicness of public spaces in traditional settlements

**DOI:** 10.1371/journal.pone.0332755

**Published:** 2025-09-30

**Authors:** Ziyang Wang, Kang Sheng, Datong Li, Tianjiao Man, Xin Zhang, Yiwen He, Qixuan Zhou, Guojing He

**Affiliations:** 1 Changxin International College of Art, Yunnan University, Kunming, China; 2 Harbin Institute of Technology (Shenzhen), School of Architecture, Harbin, China; 3 School of Architecture and Art, Central South University, Changsha, China; 4 College of Civil Engineering, Central South University of Forestry and Technology, Changsha, Hunan, China; Universiti Kebangsaan Malaysia, MALAYSIA

## Abstract

In the process of rapid urbanization in China, the public spaces of traditional settlements are undergoing significant transformations and facing numerous challenges. Systematically assessing their publicness and improving spatial quality have become critical issues. This study employs the space syntax method and Analytic Hierarchy Process (AHP) to assess the publicness of the public spaces in Zengchong Dong Village and Langde Miao Village, two traditional settlements in the Qiandongnan region, China. Drawing on field research and questionnaire data, we constructed an evaluation index system for publicness from both subjective and objective perspectives, encompassing five dimensions: accessibility, visibility, functionality, iconicity, and inclusiveness. The results show that: 1) The publicness of public spaces varies regionally, with riverside areas exhibiting higher publicness and more vibrant activities compared to adjacent mountainous areas; 2) Validation tests confirmed system reliability (R^2^ = 0.832) between calculated publicness scores and expert rating; and 3) Residents’ living habits and the differences in urban-rural perception are the main factors affecting the evaluation of public space publicness. On this basis, our study suggests building unique facilities, involving multiple parties in governance, and boosting cultural exchanges. These steps aid in reviving traditional village spaces, backing rural tourism and spurring economic and cultural growth.

## 1. Introduction

Traditional settlements, as significant components of cultural heritage, contain a rich history, unique ethnic customs, and important regional and historical significance [1]. As an indispensable type of landscape space in traditional settlements, public spaces, such as ceremonial plazas, ancient tree communities, transportation bridges, and cultural shrines, have been further expanded and enriched in terms of their spatial forms and functions along with the vigorous rise of rural tourism [2,3]. With the rise of rural tourism in China, these spaces have evolved beyond their original community functions, becoming key attractions for external visitors and important arenas for cultural exchange and economic development. However, accelerated urbanization has precipitated a sustained exodus of youth labor forces to metropolitan centers [4], resulting in the progressive dissolution of folk customs and the physical degradation of settlement public spaces. This decline has become a global challenge, threatening the sustainability and cultural vitality of traditional settlements [5,6].

As the defining characteristic of public spaces, publicness fundamentally constitutes their essential quality [7,8], serving as a critical metric for evaluating spatial quality. Scholars have studied the concept of publicness from different perspectives, confirming its importance in enhancing the quality of traditional public spaces [9]. However, in China’s ethnic minority regions, this critical attribute manifests substantial inter-settlements variations, posing significant challenges for governmental agencies in formulating context-sensitive preservation and regeneration strategies [10]. The prevalent tendency toward standardized intervention approaches frequently results in homogenized outcomes incongruent with localized socio-cultural requirements (e.g., living habits), necessitating the development of comprehensive assessment system that can continuously adapt to evolving societal demands.

Currently, research on public spaces in traditional settlements within the field of landscape architecture primarily falls into two categories. The first approach employs qualitative analysis through ethnographies and interviews [11], which emphasizes the subjective perception [12]. The second approach utilizes quantitative evaluations based on remote sensing data [13], typically focusing on the objective measurement of macro-scale spatial patterns [14]. While these methodologies have contributed human-centered perspectives and macro-scale observations, the assessment of public spaces in traditional settlements presents complex challenges that involve residents’ behavioral patterns and unique geographical constraints [9,15]. Additionally, microscale studies remain scarce, despite the critical role these public spaces play in manifesting the core attributes of traditional settlements-openness, inclusiveness, and social vitality [9]. This research gap highlights the urgent need to develop a novel framework for systematic evaluation that bridges the divide between subjective and objective assessments, integrating both qualitative and quantitative dimensions.

This study investigates public space evaluation in traditional ethnic villages through an integrated approach combining spatial analysis and social perception. Focusing on two nationally-preserved ethnic villages in the Qiandongnan region, China, we developed a comprehensive assessment framework by integrating fieldwork investigations with questionnaire surveys. The main contributions of this study are as follows: 1) Identifying key evaluation indicators of publicness, including accessibility, visibility, functionality, iconicity, and inclusiveness; 2) Integrating the space syntax method and questionnaires to enable precise measurement of subjective perception and objective assessment of public spaces; 3) Development a weighted assessment framework for publicness in traditional settlements based on the Analytic Hierarchy Process (AHP); and 4) Spatial differentiation analysis revealing distinct patterns of publicness distribution, accompanied by evidence-based optimization strategies for cultural landscape conservation.

This research establishes methodological foundations for enhancing spatial quality while preserving cultural authenticity, offering both theoretical frameworks and practical strategies for sustainable settlement development.

## 2. Related research

### 2. 1. Publicness assessment and research gaps

The concept of ‘publicness,’ originating in Western political science as a metric for evaluating governmental activities and civic values [16], has evolved into a multidimensional cultural-historical construct with discipline-specific interpretations. Political scientists emphasize democratization mechanisms [17], geographers examine place identity versus spatial anonymity [18,19], while anthropologists focus on socio-historical constructions of space. This theoretical expansion into urban studies established a public-private continuum framework [20], further developed through Habermasian notions of accessible civic forums for free discourse [21,22]. While these pluralistic perspectives offer valuable insights for China’s transitional public sphere governance, their operationalization faces empirical challenges: a persistent theory-practice gap emerges when abstract conceptualizations encounter the material realities of spatial experience [23]. Direct application of existing frameworks risks context-specific limitations, necessitating scenario-sensitive adaptations that translate theoretical dimensions into comparable metrics while preserving cross-context analytical validity [24].

Building on these theoretical foundations, empirical studies have operationalized publicness assessment through various models, including Star Model [25], Public Space Index [26], and Six-Axial Framework [27], serving as foundational frameworks for subsequent urban space evaluations. Contemporary research demonstrates multidimensional integration, exemplified by the Publicness Evaluation Model (PEM) decoding spatial evolution through urban life-physical design-governance interactions, and the Public Spaces Experience Index (PSEQI) constructing user perception metrics centered on comfort and vibrancy [9]. Empirical studies have confirmed open space quality as surpassing ownership attributes in predicting user satisfaction [28], while space syntax analyses identify path accessibility optimization as critical for public engagement [15]. Nevertheless, current assessment systems exhibit significant urban-centric bias, limiting methodological progress through three fundamental constraints: 1) underdeveloped evaluation paradigms for traditional settlements; 2) inadequate integration of intersubjective cultural practices; and 3) persistent disjunction between quantitative metrics and qualitative dimensions within culturally-sensitive evaluation systems.

### 2.2. Objective measurement of public spaces

Space syntax has emerged as a transformative analytical framework for objectively evaluating public space quality through its unique capacity to decode the socio-spatial logic embedded in built environments [29]. By constructing topological models that quantify spatial relationships, this methodology transcends conventional subjective evaluations by systematically analyzing layout configurations, connectivity patterns, and accessibility gradients [30]. Recent applications demonstrate its particular efficacy in traditional village studies, where it enables precise measurement of publicness dimensions through objective metrics like integration values and choice parameters [31,32,33]. Empirical studies reveal its dual analytical power: Gu [34] syntactic analysis of ancient tourist towns identified optimal permeability thresholds for public space networks, while Lai et al. [35] quantitatively mapped cultural influences on village residential patterns through graph-based modeling. Comparative studies across seven ethnic villages further validated its capacity to uncover universal spatial logics governing public space formation mechanisms [36]. The methodology’s true innovation lies in its ability to overcome rural evaluation biases through three critical mechanisms: 1) neutralizing subjective assessment distortions through geometric formalization; 2) establishing cross-cultural comparability via standardized topological parameters; and 3) enabling longitudinal quality monitoring through computational simulations (de la Fuente [37]). To fully realize its potential, our study’s approach bridges the existing theoretical and empirical divide by combining syntactic models with participatory perception mapping, thereby enabling comprehensive evaluations that combine objective spatial metrics with lived cultural experiences.

### 2.3. Subjective perception of public spaces

The quality of public spaces is inherently shaped by individual behavioral characteristics, which reflect sociocultural dynamics, material conditions, and personal values [38,39]. While urban-level studies have explored linkages between spatial vitality and behaviors of residents [40] and tourists [41], particularly in parks [42], streetscapes [43,44], and digitalized “check-in” spaces [45,46], critical gaps persist in understanding traditional village contexts. Existing research predominantly prioritizes physical attributes and historical values of village public spaces while neglecting villagers’ daily activities, social interactions, and cultural practices. This oversight manifests in planning frameworks that emphasize architectural styles over activity-driven vitality and disregard age/gender-based behavioral variations, ultimately undermining cultural continuity, social cohesion, and sustainable development [47,48]. Such limitations expose a fundamental disconnect between subjective spatial experiences (e.g., identity, self-esteem) and objective evaluations (e.g., foot traffic metrics, spatial configurations). This research gap highlights the urgent need to develop a novel framework for systematic evaluation that bridges the divide between subjective and objective assessments, integrating qualitative dimensions (cultural meaning, user satisfaction) with quantitative measures (behavioral frequency, spatial analytics).

## 3. Materials and methods

### 3.1. Study area

**Fig 1:**
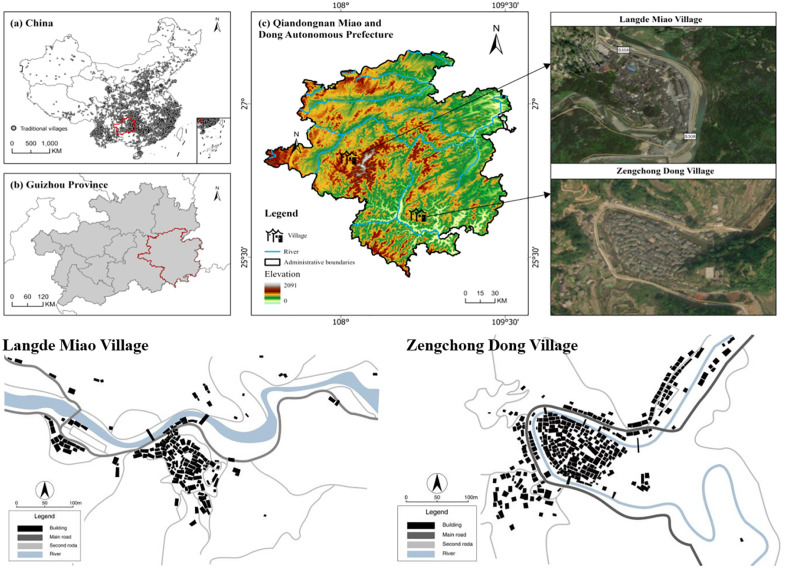
Study area.

This study focuses on traditional villages in Qiandongnan Miao and Dong Autonomous Prefecture (Abbreviation: Qiandongnan region), Guizhou Province, China, which hosts 415 traditional villages (56.8% of the provincial total), representing the country’s highest concentration of well-preserved villages with rich ecological and cultural heritage. However, widespread village “hollowing” has led to public space degradation. Selecting Zengchong Dong Village and Langde Miao Village as representative cases, this research evaluates public space dynamics in Qiandongnan’s traditional villages while controlling for ethnic heterogeneity biases (Fig 1).

**Fig 2:**
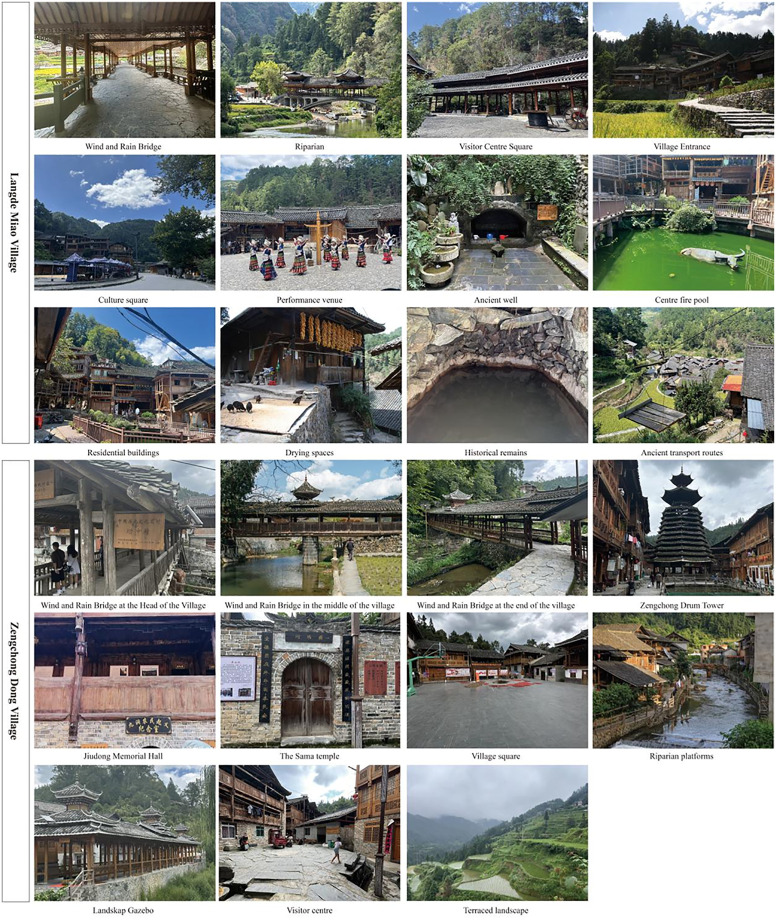
Typical public places in two villages.

Langde Miao Village (108.701°E, 25.916°N), a historic settlement 27 km southeast of Kaili City, preserves intact Miao cultural traditions through its 118 households and 500 residents. Established as one of Southeast Guizhou’s earliest ethnic tourism destinations, it attracts over 30,000 annual visitors while maintaining characteristic public spaces including the Wind-Rain Bridge and Visitor Center Square. Twelve representative public spaces were selected for analysis (Fig 2). Additionally, Zengchong Dong Village (108.067°E, 26.477°N), situated 95 km north of Congjiang County at 640 m elevation, retains traditional agricultural practices within its millennium-old Dong community of 345 households [49,50]. The village’s cultural significance is anchored by its 1672 Drum Tower, a nationally protected heritage site. Eleven characteristic public spaces, such as the Sama Temple, were examined in this study (Fig 2).

### 3.2. Methods

**Fig 3:**
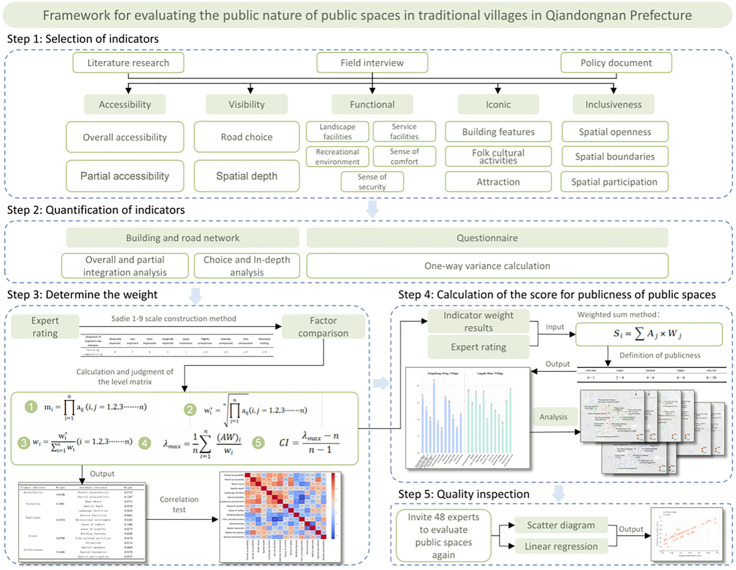
Method framework.

The method framework includes four steps (Fig 3): 1) Based on the literature review, we constructed five indicators for measuring the publicness of public spaces in traditional settlements from two dimensions: subjective perception and objective measurement; 2) These indicators were quantified through spatial analysis (i.e., space syntax) and questionnaire surveys; 3) Construction of an evaluation system through Analytic Hierarchy Process (AHP) to determine composite indicator weights; and 4) Spatial assessment using the weighted scoring method, with validation through expert panel evaluation using linear regression analysis. Based on the findings, targeted strategies for the spatial regeneration of traditional villages are proposed.

#### 3.2.1. Publicness evaluation indicators.

Through literature review, field surveys, and in-depth interviews, we employed the following methods to initially select the indicator scope for assessing the publicness of public spaces in traditional settlements: (1) compiling a systematic list of relevant research and government documents on public space quality in traditional settlements, both domestically and internationally, to develop an initial indicator framework; (2) visiting public spaces in the settlements to assess practicality, validity, and operability of the initial indicators; (3) conducting interviews with leaders of the Qiandongnan Culture and Tourism Bureau, village cadres, rural field research experts, and residents to gather perspectives on the publicness of public spaces; and (4) revising the indicator system to ensure it aligned with professional evaluation standards and the public’s needs. After eliminating irrelevant indicators and considering factors for the sustainable development of traditional settlements, we selected five indicators from different dimensions (i.e., accessibility, visibility, functionality, iconicity, and inclusiveness) to construct a public space publicness evaluation framework that integrated both subjective perception and objective measurement (Table 1).

**Table 1:** Public space publicness evaluation framework.

Dimension	Indicator	Decomposition of indicators
Accessibility	Overall accessibility	Overall road accessibility to the village.
	Partial accessibility	Some roads lead to the village.
Visibility	Spatial depth	Minimum number of connections to reach a space.
	Road selectivity	Possible road crossings.
Functional	Landscape facilities	Plants, signs, lighting and other facilities.
	Service facilities	Layout of shops, restaurants, sports and leisure facilities.
	Recreational environment	Conditions for outdoor leisure activities.
	Sense of comfort^*^	Comfort in the spatial environment.
	Sense of security^*^	User perception of psychological safety.
Iconic	Building features	The historical value of the surrounding cultural buildings.
	Folk cultural activities	Continuation of life customs and traditional folkways.
	Attraction^*^	Level of attraction of the village in terms of cultural elements and activities it represents.
Inclusiveness	Spatial openness	The extent to which villagers and others can access or use for free.
	Spatial boundaries	Spatial boundaries or dividers between different spaces or elements.
	Spatial participation^*^	Frequency of villagers’ active participation in space activities.

*represents subjective perception, quantified from questionnaire.

#### 3.2.2. Quantification of subjective and objective perceptions.

Initially, we utilized the space syntax method to measure the objective spatial characteristics of public spaces. Data on building and road networks were collected through aerial photography and on-site mapping, which were then drafted using AutoCAD. The spatial features were analyzed using Depthmap software, and an axial map was generated to assess spatial integration, choice, connectivity, and depth. Proposed by Bill Hillier in the 1970s, space syntax analyzes spatial relationships by dividing scales and segmenting spaces to reflect human movement and activity patterns [51]. It emphasizes not only local accessibility but also the comprehensibility and connectivity of the overall space. Among these, integration refers to the degree to which space in a system is clustered or discrete from other spaces. The higher the integration, the more concentrated the flow of people [52]. Choice indicates the likelihood of a path being traversed, with spaces having higher choice being more likely to be used. Depth signifies the minimum number of connections required to reach one space from another [53]. Connectivity denotes the number of spaces that intersect with a given space within the system.

In addition, we conducted a randomized survey using a questionnaire to quantify subjective perceptions of Public spaces. We conducted random surveys and interviews with villagers in Langde Miao Township and Zengchong Dong Township on two separate occasions (one weekday and one rest day) selected between 20/09/2023–30/09/2023, respectively. The surveys included demographics (gender, age, education, occupation and income), use of Public spaces, satisfaction levels and the most important indicators for measuring Public spaces. A total of 308 questionnaires were distributed in the two villages, with 300 valid questionnaires (150 per village) and a 97.4% recovery rate.SPSS analysis confirmed that the questionnaires met high standards of reliability [54]. The study was supported by Changxin International College of Arts, Yunnan University, and the research protocol was approved by the institutional ethical review board. In addition, all participants provided informed consent prior to participation. The data were credible and stable to effectively assess Public spaces.

#### 3.2.3. Determine the weight of five indicators.

This study used AHP to compare the priorities of each evaluation indicator [55]. Developed in the early 1970s by T. L. Saaty, AHP is a method for evaluating multiple objectives using network system theory [56]. It allows for convenient, flexible, and effective analysis of core issues, providing a solid foundation for decision-making. Widely used across various fields, AHP systematically analyzes different criteria levels, converts qualitative judgments into quantitative data, and accurately calculates indicator weights. In order to ensure the reliability of the results, we invited 24 experts (15 research scholars and five working designers) with extensive theoretical and practical experience in rural conservation and urban renewal, as well as four village branch cadres, to participate in the study. Each expert was asked to independently score each indicator’s importance and compare the importance differences in random pairs.

In the index structure diagram, the AHP method usually uses the product or square root methods. This study uses the square root method to calculate the influence weights of the same-level indicators. In expert scoring evaluations, the Saaty 1–9 scale construction method compares factors. By comparing the two factors, the maximum eigenvalue of the matrix and the corresponding eigenvector are calculated to construct a same-level comparison matrix. A consistency test determines the degree of influence of the indicators. The calculation and judgement process of the hierarchical matrix is as follows, based on the degree of comparability of the indicators:

(1) Multiplication of the elements of the row of the judgement matrix is performed.

$$m_{i}=\prod_{j=\text{1}}^{n}a_{ij}(i,j=\text{1},\text{2},\text{3}\cdot \cdot \cdot \cdot \cdot \cdot \cdot n)$$ (1)

(2) Square root of n.

$$w_{i}^{*}=\sqrt[{n}]{\prod_{j=\text{1}}^{n}a_{ij}}(i,j=\text{1},\text{2},\text{3}\cdot \cdot \cdot \cdot \cdot \cdot \cdot n)$$ (2)

(3) The vector $w={\left(w_{\text{1}}w_{\text{2}}\cdot \cdot \cdot \cdot \cdot \cdot w_{n}\right)}_{t}$ is normalized to obtain a weight vector.

$$w_{i}=\frac{w_{i}^{*}}{\sum_{i=\text{1}}^{n}w_{i}}(i=\text{1},\text{2},\text{3}\cdot \cdot \cdot \cdot \cdot \cdot \cdot n)$$ (3)

(4) Conduct a consistency test.

Multi-class judgment matrices generated based on multiple factors are generally positive reciprocal matrices and generally do not meet the condition of perfect consistency. Therefore, a special consistency test should be performed on them. Calculation of the maximum eigenvalue ${\;\lambda }_{max}$.


$$\lambda_{max}=\frac{\text{1}}{n}\sum_{i=\text{1}}^{n}\;\frac{{\left(AW\right)}_{i}}{w_{i}}$$ (4)

where $\lambda_{max}$ refers to the largest eigenvalue. Additionally, judgement matrix consistency index CICI (Consistency Index):


$$CI=\frac{\lambda_{max}-n}{n-\text{1}}$$ (5)

where nn refers to the matrix class. CI stands for consistency index. When CI=0, it can be guaranteed that the judgement matrix meets the criterion of complete consistency.

(5) Random consistency ratio calculation.

The criterion for evaluating the consistency of the judgement matrix CI is specified for the purpose of determining the tolerance interval for the inconsistency level of the judgement, and is introduced as the random consistency index RI. Combining the information in the above table, it can be seen that the first two classifiers with RI=0 are consistent by nature. For a multi-class classifier with n(n≥3) classes, the value of C.R. can be calculated according to Equation (6):


$$CR=\frac{CI}{RI}$$ (6)

when CR<0.1, it can be determined that the inconsistency level of the judgment matrix is generally within an allowable range. In this case, the eigenvector corresponding to the largest eigenvalue can be regarded as the weight vector of the factor. The weighted indicators shown in Table 2.

**Table 2:** Weighting of evaluation indicators.

Primary indicator	Weight	Secondary indicator	Weight
Accessibility	0. 4100	Overall accessibility	0. 2733
		Partial accessibility	0. 1367
Visibility	0. 1001	Road choice	0. 0751
		Spatial depth	0. 0250
Functional	0. 2554	Landscape facilities	0. 0428
		Service facilities	0. 0663
		Recreational environment	0. 0182
		Sense of comfort^*^	0. 1006
		Sense of security^*^	0. 0275
Iconic	0. 0700	Building features	0. 0208
		Folk cultural activities	0. 0378
		Attraction^*^	0. 0114
Inclusiveness	0. 1646	Spatial openness	0. 0869
		Spatial boundaries	0. 0230
		Spatial participation^*^	0. 0547

*represents subjective perception, quantified from questionnaire.

#### 3.2.4. Measurement and evaluation the publicness of public spaces.

Using the indicator weights obtained in the section 3.3.3 (denoted by W), the score of publicness was then calculated using the weighted sum method, according to the following formula:


$$S_{i}=\sum A_{j}\times W_{j}$$ (7)

where, $A_{j}$ represents the normalized value of the j-th indicator, and $W_{j}$ represents the weight assigned to the j-th indicator in a given space. The scores for the five dimensions are aggregated and summed to give an overall score for the publicness of a given public space, expressed as S from range of 0–10.

In addition, to validate the rationality of the proposed evaluation system, a linear regression model was introduced. Using experts’ subjective ratings of publicness as the dependent variable Y and the calculated scores from our indicators as the independent variable X, the regression equation was formulated as:


$$\begin{array}{c} Y=\beta_{\text{0}}+\beta_{\text{1}}X+\in \end{array}$$ (8)

## 4. Results

### 4.1. Overall analysis of the publicness

**Fig 4:**
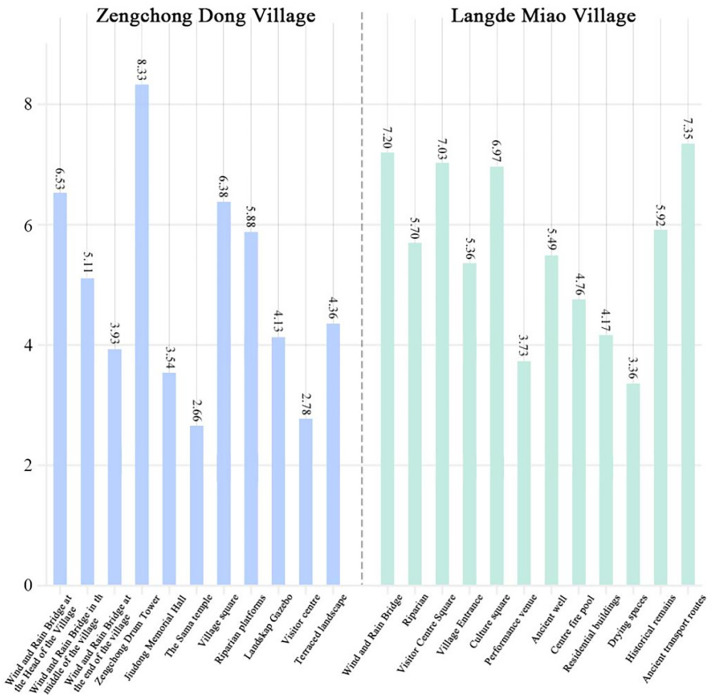
Comparison of the publicness of the 23 locations in the two villages.

According to Section 3.3.4, the publicness score for each public space in the two villages was calculated using the weighted sum of each indicator (Fig 4). In Zengchong Dong Village, the publicness score in the center of the village is generally higher than those along the river. Specifically, the publicness scores for the Zengchong Drum Tower and Village Square range from 6 to 8, indicating relatively publicness. The Landskap Gazebo, Terraced Landscape, Wind and Rain Bridge in the middle of the village, and the Riparian Platforms scored between 4 and 6, indicating ‘average’ publicness, while the Sama Temple scored 2.66, indicating low publicness. Additionally, in Langde Miao Village, the Wind and Rain Bridge, Visitor Center Square, Culture Square, and Ancient Transport Routes scored between 6 and 8, indicating high public-ness. Conversely, the Riparian, Village Entrance, Ancient Well, and Historical Remains scored between 4 and 6, indicating average publicness. The scores for Performance Field and residential buildings were in the 2–4-point range, indicating low publicness. It is worth noting that no public spaces in either village received extremely low scores, reflecting that traditional villages in the Qiandongnan region provide a high-quality public space environment for residents.

### 4.2. Verification of evaluation results

**Fig 5:**
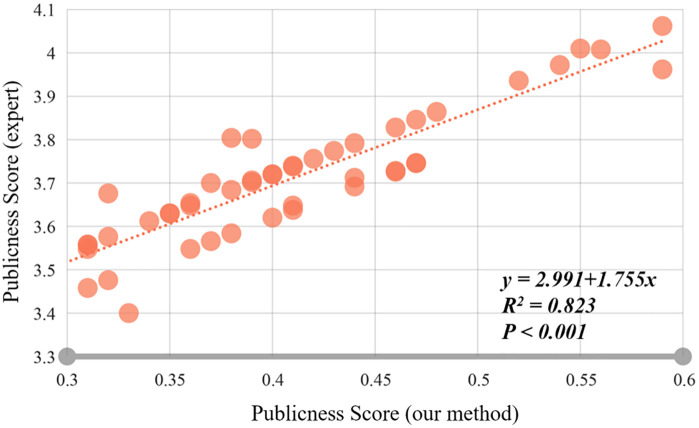
Relationship between publicness score and subjective perception score.

To ensure the objectivity, scientific rigor, and reliability of the evaluation system, 48 respondents were invited to evaluate the subjective perception of public spaces in the two villages through structured questionnaires. The respondents comprised four stakeholder groups: 12 landscape architecture experts (25%), 8 urban/rural planning scholars (17%), 10 local government officials (21%), and 18 permanent residents (38%). This stratified sampling approach ensured representation of both professional perspectives and end-user experiences. A 10-point Likert scale was adopted to quantify subjective perceptions across five dimensions: accessibility, visibility, functional, iconic, and inclusiveness quality. Scatter plots and linear regression analysis were used to examine the relationship between publicness score and expert ratings. The results shown significant linear relationship between each result, with an R^2^ = 0.823 (*p* < 0.001). Therefore, this high concordance between quantitative metrics and human evaluations confirms the validity of the publicness assessment framework (Fig 5).

### 4.3. Difference between each indicator in two villages

**Fig 6:**
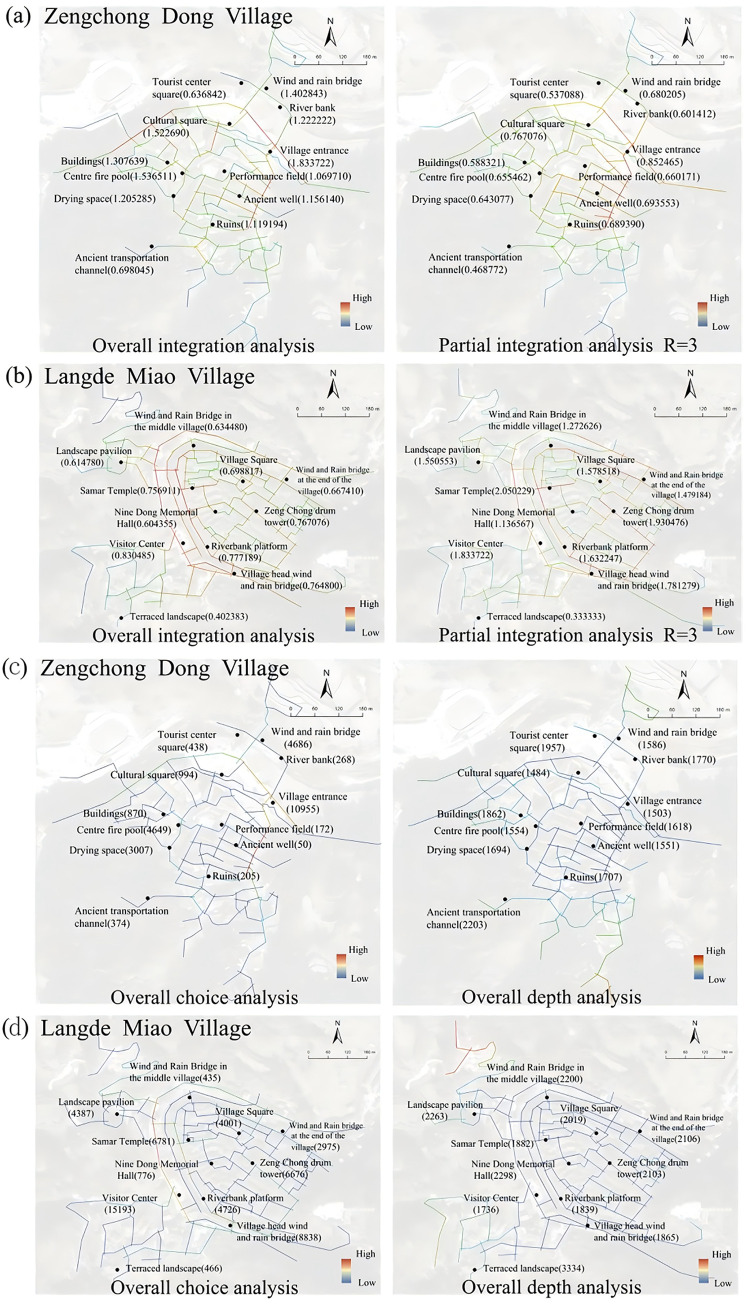
Difference between accessibility and visibility in the two villages.

The comparative analysis revealed distinct spatial patterns between Zengchong Dong and Langde Miao villages. In terms of accessibility, riverside public spaces (e.g., Visitor Center, Wind and Rain Bridge) exhibited the highest connectivity due to proximity to road networks, while mountainous areas with narrow pedestrian alleys showed reduced accessibility (Fig 6a, b). Visibility analysis further highlighted water-adjacent spaces as key nodes, particularly transport hubs like the Wind and Rain Bridge and Visitor Center, which demonstrated high choice values. Conversely, peripheral areas such as the Jiudong Memorial Hall and Terraced Landscape displayed lower visibility, reflecting their specialized roles in tourism rather than daily use (Fig 6c, d). These findings suggest a gradient of spatial utility, with core riverside areas serving as multifunctional hubs and peripheral zones accommodating niche activities.

Functional assessments indicated limited diversity across both villages, with average scores below 5 for dining, accommodation, and entertainment amenities. Zengchong’s Drum Tower emerged as an exception, excelling in worship, leisure, and security functions (Table 3, 4). However, comfort deficiencies persisted in high-security spaces like the Village Square, while remote areas like the village-end Wind and Rain Bridge scored poorly (2.99) due to inadequate seating and lighting. Langde’s historically preserved spaces (e.g., Ancient Transport Routes) outperformed newer areas like the Visitor Center Square, underscoring the impact of maintenance practices on functionality. Iconicity analysis reinforced cultural disparities: Zengchong’s seven traditional spaces scored ≥7 points across architectural and cultural dimensions, whereas Langde’s Culture Square was its sole culturally robust space, highlighting urgent needs for revitalizing spatial attractiveness in other areas (Table 3, 4).

**Table 3:** Difference between each indicator in Zengchong Dong Village.

Locations	Publicness indicators				
	Accessibility	Visibility	Functional	Iconic	Inclusiveness
Wind and Rain Bridge at the head of the village	7.46	4.15	5. 08	7. 34	7. 59
Wind and Rain Bridge in the middle of the village	5.50	2.94	3. 09	7. 09	7. 78
Wind and Rain Bridge at the end of the village	3.54	1.73	2. 99	7. 10	6. 33
Zengchong Drum Tower	8.76	7.27	8. 04	9. 05	8. 04
Jiudong Memorial Hall	2.79	1.49	4. 54	5. 66	4. 19
The Sama Temple	1.89	2.27	2. 87	5. 80	3. 14
Village Square	7.93	6.26	5. 89	3. 40	4. 61
Riparian Platforms	5.97	5.02	7. 26	3. 22	5. 14
Landskap Gazebo	3,35	3.25	6. 65	2. 21	3. 55
Visitor Center	1.65	3.23	4. 32	2. 86	2. 91
Terraced Landscape	2.23	5.27	4. 20	6. 47	8. 21

**Table 4:** Difference between each indicator in Langde Miao Village.

Locations	Publicness indicators				
	Accessibility	Visibility	Functional	Iconic	Inclusiveness
Wind and Rain Bridge	8.04	6.65	6. 59	3. 16	8. 07
Riparian	5.63	4.94	5. 75	4. 79	6. 64
Visitor Center Square	8.63	7.23	3. 53	6. 51	8. 59
Village Entrance	6.46	5.52	3. 67	4. 62	5. 45
Culture Square	6.79	5.99	6. 85	8. 90	7. 41
Performance Field	3.89	6.02	2. 47	4. 72	3. 51
Ancient Well	5.93	7.51	4. 39	4. 30	5. 35
Centerfire Pool	5.97	5.27	3. 14	4. 59	4. 04
Residential buildings	5.01	3.00	2. 65	6. 25	4. 24
Drying Spaces	3.99	1.98	2. 74	4. 91	2. 96
Historical Remains	2.67	4.27	5. 23	7. 20	7. 48
Ancient Transport Routes	8.31	6.66	6. 67	3. 40	8. 12

Inclusiveness varied significantly between villages. Open, barrier-free spaces like Zengchong’s Drum Tower and Langde’s Wind and Rain Bridge demonstrated high accessibility and public participation. However, restricted spaces such as Sama Temple (openness: 3.38) and Center Fire Pool (participation: 6.33) revealed mismatches between physical access and social inclusion (Table 3, 4). These findings emphasize that optimizing traditional village public spaces requires simultaneous improvements in spatial permeability, cultural vitality, and participatory design to balance functional utility with community engagement.

### 4.3. Human behaviors explain the differences in publicness

**Fig 7:**
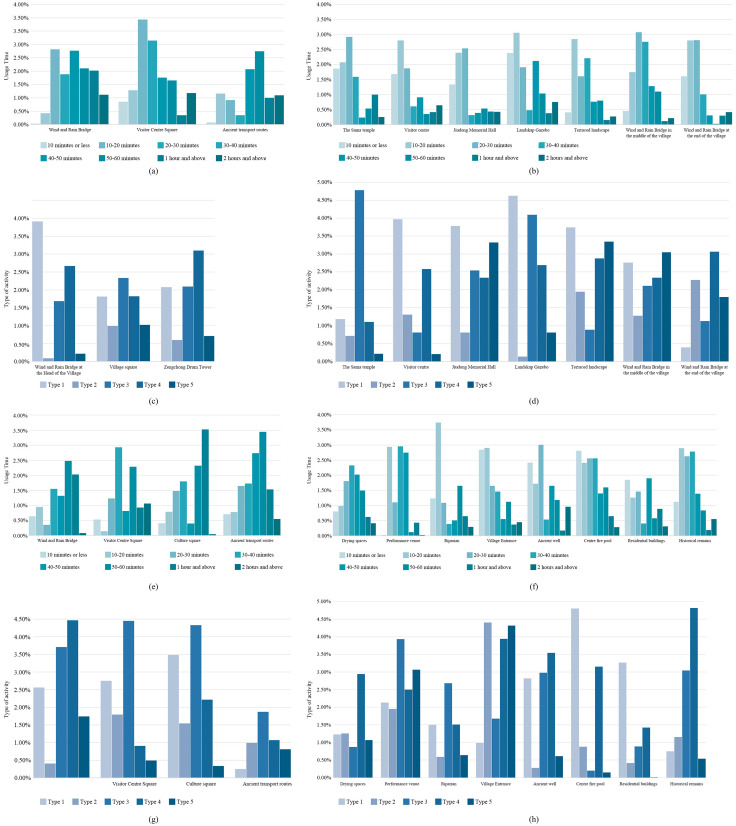
Percentage of public spaces and types of activities: (a) Time spent in highly public spaces in Zengchong Dong Village; (b) Time spent in moderately and less public spaces in Zengchong Dong Village; (c) Types of activities in highly public spaces in Zengchong Dong Village; (d) Types of activities in moderately and less public spaces in Zengchong Dong Village; (e) Time spent in highly public spaces in Langde Miao Village; (f) Time spent in moderately and less public spaces in Langde Miao Village; (g) Types of activities in highly public spaces in Langde Miao Village; (h) Types of activities in moderately and less public spaces in Langde Miao Village.

Additionally, based on the results of the previous questionnaire survey, we provided evidence of the impact of human behavior on the publicness of different public spaces from the perspective of human behavior. Specifically, we found that in Zengchong Dong Village, over 50% of the villagers spend no more than 2 hours in public spaces, with more than 80% engaging in 2–3 types of activities, mainly leisure, shopping, and social interaction (Fig 7c, d). In highly public spaces (Fig 7a), such as the Village Square and Wind and Rain Bridge at the head of the Village, villagers typically linger for 30–50 minutes, with some choosing to stay for 1–2 hours or longer at the Zengchong Drum Tower. In contrast, in spaces with medium to low publicness (Fig 7b), such as the Landskap Gazebo and the Wind and Rain Bridge in the middle of the village, visits are shorter, usually 10–30 minutes. This reveals that cultural factors may influence the limited time villagers spend in these areas. In addition, 48.67% of the villagers engaged in only one type of activity, primarily sitting or chatting, while participation in two or more activities decreased significantly. In the Village Square and Zengchong Drum Tower, villagers commonly participated in 3–4 activities, whereas in places such as the Landskap Gazebo, Sama Temple, and Wind and Rain Bridge at the head of the village, activities are predominantly singular (Fig 7c, d).

In Langde Miao Village, only 20% of villagers spend less than 2 hours in public spaces. Among them, 80% engage in 3–4 types of activities (Fig 7g, h), mainly social interaction and leisure. In high-level public spaces (Fig 7e), villagers are more inclined to stay for 50–60 minutes or longer, while in medium to low-level public spaces (Fig 7f), they typically stay for less than 30 minutes. Moreover, more than 60% of villagers engaged in multiple activities, such as exercising, sunbathing, and caring for children. Although public spaces in Zengchong Dong Village and Langde Miao Village show a high degree of consistency in their public scores, significant differences exist in the types of activities occurring within them. Comparing to previous research results, this difference is closely related to the distinct ethnic backgrounds of the two villages.

## 5. Discussions

### 5.1. Influencing factors of publicness in traditional settlements

The study reveals significant heterogeneity in public space publicness across villages within identical geographical regions, echoing the comparative framework proposed by Yaylali-Yildiz et al. [57] for campus spaces. Three interrelated barriers emerge: (1) Geomorphological constraints in narrow or hilly terrains fragment transportation networks, substantially reducing accessibility to village cores. This spatial dispersion aligns with the connectivity theory developed by Barnes et al. [58], where villages with radial road systems demonstrated enhanced public engagement. (2) Functional homogenization diminishes spatial vitality, as most surveyed spaces lacked social-cultural activities. This finding supports the behavioral constraints framework articulated by Kabeer [59], where mono-functional spaces systematically excluded elderly and youth users. (3) Social interaction deficits weaken community cohesion, particularly evident in villages with low community spirit metrics. Case studies demonstrate that sports infrastructure integration strategies [60,61] and landscape water features significantly increased cross-generational interactions. Spatial optimization should prioritize transport-network integration, functional diversification through participatory design, and heritage-adaptive social infrastructure.

**Fig 8:**
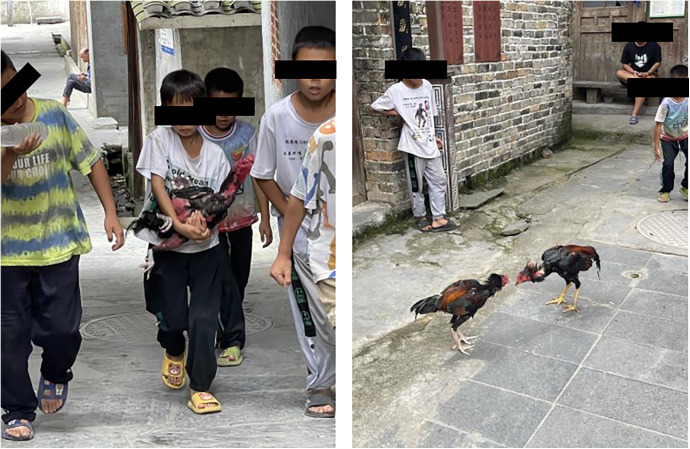
Children playing ‘cockfighting’ in Zengchong Dong Village.

Age-stratified analysis uncovers divergent evaluation patterns: elderly residents exhibited stronger satisfaction compared to middle-aged adults, consistent with findings on urban-rural nostalgia reported by Zhao et al. [62]. The spatial attachment of seniors showed strong correlation with heritage conservation completeness and walkability metrics emphasized in studies by Scannell and Gifford [63], Casakin et al. [64], though underdeveloped villages displayed markedly reduced elderly mobility due to inadequate facilities. Youth evaluations revealed pronounced preference for recreational spaces supporting peer socialization, yet rural youth consistently reported limited access compared to urban counterparts as documented by Poitras et al. [65]. Life experience and social role emerged as dominant subjective predictors, mediated through environmental comfort perception. Field observations validated that villages maintaining regular communal events fostered stronger intergenerational place attachment through ritualized interactions (Fig 8).

### 5.2. Mechanisms of the publicness of public spaces in traditional settlements

Traditional villages exhibit distinct public space configurations compared to urban areas, particularly in openness and functionality. Village spaces maintain ambiguous boundaries where communal and private realms interpenetrate, as seen in courtyard systems that organically integrate agricultural activities with social interactions [66]. This contrasts with urban spaces like parks and libraries that enforce clear public-private demarcations. Spatial features such as vernacular paving styles and ritualized planting patterns reinforce villagers’ place attachment [67], while their functionality centers on cultivating social cohesion through shared agrarian practices and collective decision-making [68]. Unlike cities’ specialized recreational facilities, village spaces operate as hybrid platforms embedding labor, cultural transmission, and community governance.

Tourism-driven spatial transformations are redefining ownership structures in traditional villages. The shift from agrarian to commercial-tourism economies has generated hybrid “production-living-ecological” spaces [69], where ancestral lands now accommodate merchant stalls and guesthouses alongside heritage sites. This transition introduces ternary stakeholders-villagers, tourists, and entrepreneurs-whose competing claims create fluid ownership patterns. While ecological protection efforts attempt to balance development, the commodification of cultural landscapes risks displacing endogenous social networks (Felipe [70]). Nevertheless, these hybrid spaces demonstrate adaptive potential by integrating economic activities with traditional collectivism, albeit requiring careful governance to sustain community agency.

### 5.3. Strategies for the renewal and protection of public spaces

Effective renewal of traditional village public spaces requires context-sensitive spatial planning and tourism integration. The “one village, one system” approach prioritizes ethnohistorical analysis through village archives and land use records, enabling customized public facility development that preserves cultural authenticity while meeting contemporary needs. This strategy counters spatial homogenization by synergizing vernacular architecture with modern functions, as demonstrated in ancestral square adaptations combining ritual spaces with craft markets. Concurrently, tourism integration transforms heritage assets like ancient theaters into cultural experience hubs, where traditional handicraft demonstrations attract visitors while generating preservation funds. Such spatial interventions achieve dual objectives: maintaining biocultural diversity through landscape-sensitive design and creating economic incentives for heritage stewardship through tourism revenue-sharing models.

Addressing governance fragmentation necessitates establishing multi-stakeholder coalitions encompassing villagers, collective economic organizations, and township authorities. The proposed tripartite governance model institutionalizes resident participation in decision-making through co-design workshops and benefit-sharing mechanisms, effectively bridging national policy implementation with local spatial needs. Complementing this structural reform, cultural revitalization initiatives reactivate public spaces as identity anchors – digital storytelling platforms installed in drum towers engage youth through augmented reality reconstructions of village history, while seasonal festivals curated with elders reinforce intergenerational knowledge transmission [71]. These efforts are operationalized through smart village systems that monitor spatial usage patterns and optimize maintenance schedules, ensuring governance responsiveness to evolving community dynamics while preventing commercial overexploitation of cultural assets.

### 5.4. Limitations

However, despite the contributions of this study to understanding of the public-ness of traditional village public spaces, several limitations remain. Due to the limited previous research in this field, the assessment indicators and their weightings may require further refinement based on specific rural contexts. The methodology could also benefit from the integration of advanced technologies, such as big data analysis, to optimize publicness assessment. Although this study included traditional villages from various ethnic groups, the small sample size may limit its representativeness, as it may not have fully capture the spatial characteristics of all villages. To overcome this limitation, future research should expand the scope of this study and refine the indicator weightings for a more comprehensive assessment.

## 6. Conclusion

This study develops a comprehensive evaluation system for public space publicness in traditional villages, using Zengchong Dong and Langde Miao Villages in Qiandongnan as case studies. The framework integrates 5 dimensions and 15 indicators, analyzed through field surveys, questionnaires, and space syntax. Key findings reveal accessibility metrics (overall and partial) as the most influential factors, followed by comfort and spatial openness. Cultural landmarks like Drum Towers and Wind-Rain Bridges demonstrated exceptional inclusivity, while historically significant multifunctional spaces near mountainous areas showed superior visibility compared to river-adjacent locations. Validation tests confirmed system reliability (R^2^ = 0.832) between calculated publicness scores and villagers’ perceptions, providing planners and policymakers with evidence-based optimization strategies.

The research highlights significant regional disparities in public space quality, with over 60% of surveyed areas scoring low in publicness. It uncovers an urban-rural perceptual divide in spatial evaluation and emphasizes the symbiotic relationship between vernacular cultural elements and spatial layouts. These insights offer empirical support for tailoring public space enhancement strategies in ethnic villages while preserving cultural authenticity, ultimately contributing to sustainable rural development through spatially sensitive interventions.
